# Targeted conspiratorial killing, human self-domestication and the evolution of groupishness

**DOI:** 10.1017/ehs.2021.20

**Published:** 2021-04-06

**Authors:** Richard W. Wrangham

**Affiliations:** Department of Human Evolutionary Biology, Harvard University, Cambridge, MA, USA

**Keywords:** Execution hypothesis, morality, cooperation, chimpanzee

## Abstract

Groupishness is a set of tendencies to respond to group members with prosociality and cooperation in ways that transcend apparent self-interest. Its evolution is puzzling because it gives the impression of breaking the ordinary rules of natural selection. Boehm's solution is that moral elements of groupishness originated and evolved as a result of group members becoming efficient executioners of antisocial individuals, and he noted that self-domestication would have proceeded from the same dynamic. Self-domestication is indicated first at ~300,000 years ago and has probably gathered pace ever since, suggesting selection for self-domestication and groupishness for at least 12,000 generations. Here I propose that a specifically human style of violence, targeted conspiratorial killing, contributed importantly to both self-domestication and to promoting groupishness. Targeted conspiratorial killing is unknown in chimpanzees or any other vertebrate, and is significant because it permits coalitions to kill antisocial individuals cheaply. The hypothesis that major elements of groupishness are due to targeted conspiratorial killing helps explain why they are much more elaborated in humans than in other species.

## Graphical abstract

**Figure:**
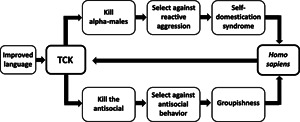

**Social media summary:** Planned killing of antisocial individuals occurs only in humans, and helps to explain why we have prosocial tendencies.

Unlike other vertebrates, humans (*Homo sapiens*) have long been considered to be more cooperative and prosocial than expected from theories of kin selection or mutualism. One kind of explanation credits humans’ elaborate cognitive abilities, because these permit more complex systems of reciprocal exchange than other animals can manage, including social norms, contracts and laws (dos Santos & West, 2018). In addition to such proximate pressures, however, evidence of prosociality in human infants suggests that human tendencies to be more cooperative than other primates are evolved traits (Graham et al., 2013; Tomasello, 2016). Furthermore human prosociality has genetic and neuroanatomical correlates (Raghanti, 2019; Tiihonen et al., 2020). Following Haidt (2012) I call such evolved propensities ‘groupishness’, characterised as a tendency to cooperate and be prosocial in ways that appear to transcend genetic self-interest. Groupishness in humans includes spontaneously helping unrelated group members, having a social conscience, accepting and enforcing a moral code, conforming to group norms, sharing resources, and being concerned about fairness and reputation. In this paper I suggest that the evolution of human groupishness was strongly influenced by a unique human ability, targeted conspiratorial killing.

Groupishness is a conundrum because individuals are expected to be self-interested except when they can sufficiently benefit kin. Darwin (1871) grappled with this problem when trying to explain the evolution of morality. He assumed that individuals who aid non-kin experience a cost relative to those who are less prosocial, and therefore concluded that such behaviour was not explicable in terms of natural selection theory acting within groups. Most subsequent investigators have reached the same impasse, leading some to invoke human-specific applications of group selection or multilevel selection theory (e.g. Darwin, 1871; Choi & Bowles, 2007; Wilson &Wilson, 2007). Such efforts are generally considered unsuccessful (West et al., 2011).

An alternative approach challenges the core assumption that agents suffer costs by being groupish. Instead, groupishness benefits agents by protecting them from punishment. The argument depends on a special feature of human society. Within groups, individuals with a reputation for being antisocial can be punished by coalitions of others. If such punishments are sufficiently systematic and costly, groupishness is less costly than selfishness. According to this perspective groupishness is equivalent to a self-imposed tax. The tax protects the agent from the long-term costs of acting against the interests of the punishing alliance (Boehm, 2012; Wrangham, 2019b).

Darwin (1871) noted that selection acts against aggressiveness when violent men are executed or imprisoned, but he did not pursue the implications of this observation. More than a century later Boehm (1999, 2012, 2014, 2017, 2018; Gintis et al., 2015) argued explicitly that groupishness benefits individuals because in hunter–gatherer societies that represent an environment of evolutionary adaptedness, the costs of antisocial behaviour can be very high. Boehm's focus, like Darwin's, was on the morality of fairness, a major component of groupishness.

Moral feelings associated with fairness were once thought to be present in non-humans such as capuchins (*Cebus apella*) and chimpanzees (*Pan troglodytes*) (Brosnan & de Waal, 2003). Experiments show, however, that only humans have a tendency to sacrifice personal gain for the sake of equality, whereas non-humans’ apparent concern for fairness reflects other motivations such as efforts to manipulate an experimenter (Engelmann et al., 2017; McAuliffe & Santos, 2018). Accordingly traits associated with fairness such as senses of responsibility, obligation, duty, guilt and shame appear to be restricted to humans, making their evolution a particularly interesting puzzle (Tomasello, 2016). In contrast moral emotions concerned with sympathy, such as compassion, concern and benevolence, are evidenced in non-humans (de Waal, 2006).

According to Boehm a mid-Pleistocene phase of distinctive moral evolution began in *Homo* as a result of alliances of bullied males predictably killing unremittingly aggressive alpha males within their own group. The fact that such an alliance could safely dispatch the most physically intimidating member of the group meant that it could equally well kill any other group member. Accordingly a wide set of antisocial behaviours became intensely risky for group members, such that a reputation not merely for being a violent bully but also for being a trouble-maker, competitor, bringer of bad luck or consistently selfish could lead to an individual being killed. This novel threat of the severest punishment for antisocial behaviour created a strong incentive for following norms for the sake of self-protection, provided that the costs of doing so were not too high. The long-term result was selection against antisocial behaviour and in favour of prosocial behaviour, cooperation and conformism, a dynamic that ultimately favoured the moral senses and other components of groupishness. In short, groupishness was favoured when the evolution of capital punishment meant that selfish behaviour became much more costly than previously (Cofnas, 2018; Wrangham, 2019b).

Boehm's ‘execution hypothesis’ has several merits. It fits the historical record of every kind of society, from small-scale to state, because executions have been a conventional mechanism of controlling antisocial and amoral behaviour worldwide (Woodburn, 1982; Otterbein, 1986; Boehm, 1999, 2017). It provides a logical explanation for how despotic alpha-male behaviour, which is typical of group-living primates, was controlled and selected against in *H. sapiens* (Boehm, 1999, 2012; Wrangham, 2019a). It accounts for males in small-scale societies having egaIitarian social relationships in the form of a reverse dominance hierarchy (Boehm, 1993; Erdal & Whiten, 1994). It fits the inference that Pleistocene *Homo* were skilled killers of large animals, suggesting that well-planned proactive kills of group members would have been low risk. The idea that Pleistocene *Homo* were capable of within-group killing is also supported by the fact that coalitions of chimpanzees are likewise known to kill adults within their own groups, reportedly including especially aggressive individuals (Boehm, 2012; Wilson et al., 2014). The hypothesis that a violent form of social selection could have been responsible for promoting the evolution of groupishness thus fits data on both humans and chimpanzees.

The execution hypothesis suffers, on the other hand, from at least three kinds of problem. First, theoretical models have not identified the conditions under which subordinates benefit by proactively killing the alpha. This omission is problematic because in some circumstances subordinates could in theory benefit from the presence of an alpha that contributes positively to collective actions (Gavrilets & Fortunato, 2014; Gavrilets, 2015). Here, in contrast to that idea, I assume that if subordinates’ cognitive ability allows them to kill a selfish alpha cheaply, and so proactively that the alpha has no opportunity to form a counter-coalition, subordinates always benefit from killing him or her. Although this idea is derived from the ethnographic record (Boehm, 2012), and in spite of some relevant theorising (Gavrilets et al., 2008; Gavrilets, 2012; Ihara, 2020), the conditions for it to be true do not appear to have been formally modelled (Mesterton-Gibbons et al., 2011; Bissonnette et al., 2015).

Second, the execution hypothesis is difficult to test because the frequency and patterning of Pleistocene executions is unknown. When and how rapidly groupishness was influenced by the proposed selection pressure therefore cannot be estimated directly. The evolution of groupishness is logically linked to the evolution of self-domestication, however, by the claim that both outcomes result from the same selection pressure against highly aggressive behaviour, namely targeted killing (Boehm, 2012, 2017; Hare, 2017; Wrangham, 2019b; see Figure 1). This inferred linkage is useful because unlike groupishness, self-domestication has anatomical and genetic correlates that indicate the pattern of its evolution. Inferrable features of the evolution of self-domestication can therefore in theory be used to inform an understanding of the evolution of groupishness.

**Figure 1.**
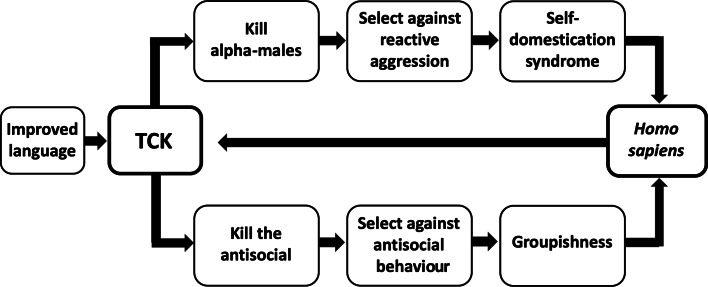
Hypothesised effects of targeted conspiratorial killing (TCK). The upper and lower pathways diagram the evolution of self-domestication and groupishness, respectively. Targeted conspiratorial killing is cheap because it can be conducted at low risk to the killers. It was putatively enabled by language becoming sufficiently sophisticated to foster conspiratorial behaviour. A positive feedback loop followed as a result of increases in features such as social tolerance, linguistic skills and tendencies for conformity, which made targeted conspiratorial killing increasingly easy to organise.

The third weakness to be discussed is the problem of why within-group killing had different evolutionary results in chimpanzees from *H. sapiens* despite occurring in supposedly similar ways. The ideal solution will explain not only why groupishness occurs in humans, but also why it is found barely or not at all in chimpanzees or other non-humans. I consider four suggestions.

The first is morality. Boehm (2017, p. 761) proposed that morality made within-group killing more effective in humans than in chimpanzees: ‘a human group consumed with moral outrage can become a still more efficient killing machine’. While this idea may well be correct, it does not fit Boehm's core argument that within-group killing was responsible for the evolution of human-style morality. If human-style morality was produced by within-group killing, something else must have given within-group killing its initial special power.

Second, Boehm (2014, p. 170) alluded to the possession of lethal weapons as a reason why ‘we have taken our own evolutionary course’ compared with chimpanzees. The idea that the use of weapons transformed human social life has been suggested often since Woodburn (1982) pointed out that well-armed men such as hunter–gatherers can easily kill other members of their groups (Bingham, 2000; Okada and Bingham, 2008; Phillips et al., 2014; Chapais, 2015; Gintis et al., 2015). For weaponry to have been critical, the origin of a newly efficient type of lethal weapon should have coincided with, or immediately pre-dated, the purported origin of self-domestication and elaboration of human groupishness at ~300,000 years ago or shortly after. This means that the type of weaponry must have been an advance on spears, because spears were already sophisticated by 400,000 years ago (Thieme, 1997). Lithic culture appears to have become more complex around 300,000 years ago (Shea, 2017; Brooks et al., 2018), but complex projectile weapons are not known until 60,000–70,000 years ago (arrows, Backwell et al., 2017). The apparent lack of any significant improvement in weaponry coinciding with the earliest evidence of self-domestication undermines the idea of its importance in the evolution of groupishness.

Third, Boehm (2012) argued that climate change in the middle Pleistocene was a critical factor. His idea was that unfavourable weather made food production, and especially meat acquisition, unpredictably hazardous. In these circumstances, he suggested, food-sharing became necessary, and despots who refused to share meat were killed so that everyone could survive. In support of this proposal, Boehm (2014) cited archaeological evidence that between 400,000 and 250,000 years ago patterns of hunting and meat-sharing changed to reflect a greater importance of cooperation. The climate explanation treats the cognitive ability of mid-Pleistocene *Homo* and chimpanzees as being equally well adapted for within-group killing, that is, climate variation merely elicited a ‘potential [that] has been continuously present in the human, chimpanzee, and bonobo lines for five to seven million years’ (Boehm, 2018, p. 696). Evidence reviewed in this paper will challenge that notion however, because chimpanzees appear to be incapable of organising the killings of despots.

Fourth, Wrangham (2019a, b) proposed that the style of within-group killing is critically different between humans and chimpanzees because only humans have enough language ability to plan executions to be cheap and safe for the killers.

Below, I investigate why within-group gang attacks have favoured groupishness in humans but not in chimpanzees. I begin by considering the relationship between groupishness and self-domestication.

## Self-domestication

Scholars have claimed for more than 2000 years that humans are behaviourally similar to domesticated animals (Wrangham 2019b). Until Darwin, the assumption was that humans had always been domesticates, but when evolutionary theory replaced natural theology the implication changed. Similarities between humans and domesticated animals then became attributed to humans having evolved from a less domesticated ancestor (Bagehot, 1872).

Boas (1911) found the idea irresistible and was the first to call humans ‘self-domesticated’. He suggested that self-domestication began in the early Quaternary, at the start of the Pleistocene, and was further developed after fire was controlled (Boas, 1911, p. 76). The self-domestication idea duly gained traction (review by Brüne, 2007; e.g. Fischer, 1914; Lorenz, 1940; Mead, 1954; Gould, 1977; Belyaev, 1984). However, against the notion, the criteria for deciding whether humans qualified as domesticates were vague, and no mechanism was proposed for why self-domestication might have occurred. The idea did not appear to be testable, and the concept of self-domestication seemed weirdly particular because it was applied only to humans and not to any other species. For such reasons the idea was often dismissed (Darwin, 1871; Haldane, 1956; Dobzhansky, 1962). In the last 20 years, however, self-domestication theory has overcome many of these problems.

The meaning of self-domestication is an important issue that requires consideration of the meaning of ‘domestication’ also. Traditional definitions of domestication often include criteria such as one species taking care of another, one species benefiting from the relationship with another species, or the domesticate living as a pet or on a farm (Leach, 2003; Zeder, 2015). To include such criteria in a definition of domestication would make the concept of self-domestication meaningless.

Theorists of human self-domestication therefore define both domestication and self-domestication in terms of their biological consequences (Thomas & Kirby, 2018; Wrangham, 2019b). All of the many definitions of domestication invoke the resulting relationship between domesticator and domesticate (Zeder, 2015). Tameness is the *sine qua non* of this relationship, and is the only domestication-related trait known to occur in all domesticated mammals and birds (Lord et al., 2020; Sánchez-Villagra & van Schaik, 2019). Much evidence indicates that selection for tameness is primarily responsible for domesticated traits in general (Arbuckle, 2005; Wilkins et al., 2014; Trut et al., 1991, 2020; Zeder, 2020). For those reasons domestication and self-domestication are conveniently defined purely in terms of tameness, equivalent to docility or a reduced propensity for reactive aggression (Wrangham, 2018).

Here therefore I define both domestication and self-domestication as a reduction in a species’ propensity for reactive aggression. Farmyard domesticates tend to exhibit reduced reactive aggression towards both humans and conspecifics. In self-domesticated species, in contrast, the reduction in the propensity for aggression occurs without any other species being actively engaged, and the aggression being reduced is primarily towards conspecifics (Hare et al., 2012; Hare, 2017; Theofanopolou et al., 2017; Wrangham, 2019b). However self-domestication can in theory reduce aggression towards humans, as suggested for urban animals (Hare and Woods, 2020; Parsons et al., 2020).

Domestication and self-domestication are thus different processes, but the self-domestication hypothesis proposes that they share important underlying commonalities in biological mechanisms. Most importantly, selection against reactive aggression is predicted to generate a similar syndrome of traits in each case. This concept of a domestication or self-domestication syndrome does not mean that all traits in the syndrome invariably co-occur: they do not (Lord et al., 2020). Instead, it means that they occur with statistical regularity (Wilkins et al., 2014; Wilkins, 2017; Trut et al., 2020; Zeder, 2020), regardless of whether the selective pressure comes from human agency or from any of a variety of mechanisms in the wild that favours reduced reactive aggression (Hare et al., 2012; Hare, 2017; Wrangham, 2018, 2019b).

Note that a definition of self-domestication in terms of selection for reduced aggressiveness means that self-domestication must have happened often in the history of life. Yet the first example of self-domestication in a wild animal was not proposed until 2012 (bonobos *Pan paniscus*; Hare et al., 2012). Two reasons could explain why self-domestication has been detected rarely.

First, the ability to recognise it depends on comparison between a species that has experienced selection against aggression and a closely related species that has not. However, the required comparand will often not exist. In the absence of an informative relative of a giraffe, we have no way to infer the evolutionary history of giraffes’ aggressiveness.

Second, as expected with antagonistic pleiotropy, signals of self-domestication that occurred long ago are expected to be masked by subsequent evolutionary changes (Stearns & Medzhitov, 2015). Losses would include non-adaptive traits originally produced as part of a self-domestication syndrome.

The best opportunities for recognising that a species self-domesticated will therefore be when the target species has a close relative or a palaeontological record that helps to model the unselected ancestor, and when self-domestication has been recent. The frequency of such conditions has not been estimated. Here I briefly consider self-domestication in non-humans to support the claim that selection against reactive aggression in the wild can have parallel effects to those found in captivity.

## Self-domestication in non-humans

The most closely examined case for self-domestication in a wild species is for bonobos (Hare et al., 2012). Bonobos and chimpanzees are sister species separated by the Congo River ever since a dry period allowed their common ancestor to cross from the right bank to the left, where bonobos now live (Takemoto et al., 2015; de Manuel et al., 2016). Separation occurred between 0.87 (Won & Hey, 2005) and 2.1 million years ago (de Manuel et al., 2016). Gorillas (*Gorilla gorilla*) are the closest out-group to *Pan*, and chimpanzees show more similarities to gorillas than bonobos do, including in the behaviourally significant cranio-facial region. This indicates that chimpanzees have changed less from the common ancestor than bonobos have (Pilbeam & Lieberman, 2017).

**Figure 2.**
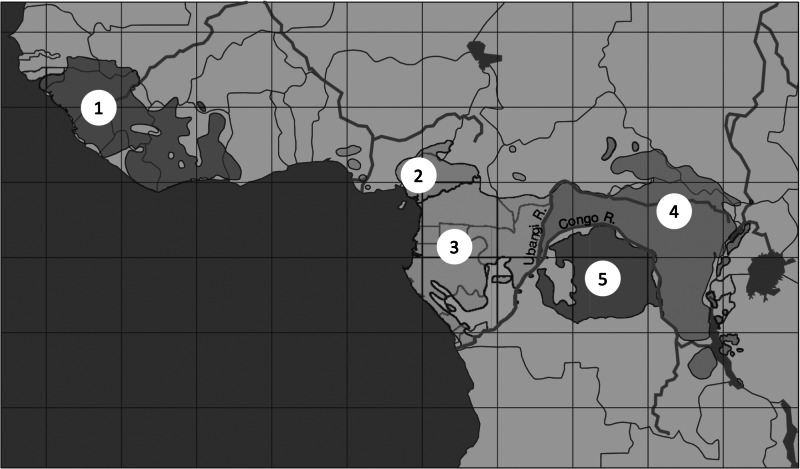
Distribution of chimpanzees and bonobos. 1, Western chimpanzee, *P. troglodytes verus*; 2, Nigerian–Cameroonian chimpanzee, *P. troglodytes ellioti*; 3, central chimpanzee, *P. troglodytes troglodytes*; 4, eastern chimpanzee, *P. troglodytes schweinfurthii*; 5, bonobo, *P. paniscus. P. troglodytes verus* is separated from *P. troglodytes ellioti* by the Dahomey Gap, a region too dry to support forest. *P. paniscus* is separated from *P. troglodytes schweinfurthii* by the Congo River. Map is from Prüfer et al. (2012).

The argument for self-domestication comes from comparing phenotypes. Relative to chimpanzees, bonobos have much less aggressive males and they exhibit many anatomical, behavioural and cognitive traits that are characteristic of domesticated animals, including short faces, smaller teeth, smaller brains, reduced sexual dimorphism in teeth, reduced body mass, increased play, increased gregariousness, increased tolerance, delayed cognitive development and neotenous crania (Hare et al., 2012; Hare, 2017; Rosati, 2019; Wrangham, 2019b). The many convergences with domesticated animals and the inferred polarity of evolutionary change suggest that the mechanisms responsible for reduced aggression in bonobos are similar to those in domesticated animals. Initial genetic tests support this hypothesis (Kovalaskas et al., 2020).

Why bonobos evolved a reduced propensity for aggression compared with chimpanzees is not known. An ecological hypothesis points to functional consequences of gorillas being absent in the bonobo range, whereas most chimpanzees that live in similar habitats to bonobos co-occur with gorillas (Hare et al., 2012). According to this idea, the absence of proto-gorillas in the proto-bonobo habitat allowed proto-bonobos to utilise ‘gorilla foods’ more and therefore to be more gregarious than their chimpanzee-like ancestors (cf. Toda & Furuichi, 2020). Increased gregariousness allowed female bonobos to form self-protective mutual coalitions in response to male aggression and to increase their ability to choose preferred males as mates; the fitness of the most aggressive males was therefore reduced. This scenario is based on the principle that the present is a key to the past, given that female bonobos are routinely seen to collaborate in defeating aggressive males (Tokuyama & Furuichi, 2016). While no tests have been devised for this ecological scenario, it illustrates the theoretical point that self-domestication is expected to occur via diverse mechanisms.

**Figure 3.**
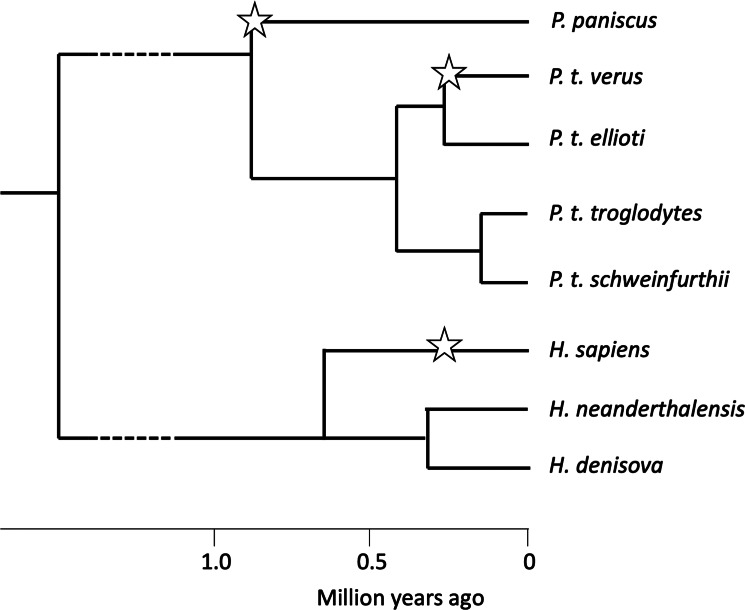
Inferred population history of *Pan* and *Homo*, showing possible self-domestication events (starred). Ape phylogeny is from Prado-Martinez et al. (2013), who estimated the times of population splits. *Homo* phylogeny is from Schlebusch et al. (2017). No fossil data are available to help estimate the times of self-domestication in *P. paniscus* and *P. troglodytes verus.*

A more speculative case of self-domestication in the genus *Pan* is suggested by research on western chimpanzees, *P. troglodytes verus*. Yamakoshi (2004) and Pruetz et al. (2017) noted that western chimpanzees are less aggressive, more tolerant and more gregarious than eastern chimpanzees, *P. troglodytes schweinfurthii*. Shea and Coolidge (1988) measured whole skulls and found that the length of the cranium as a whole, as well as the combined length of the maxillary molar row, was significantly reduced in western compared with eastern chimpanzees, as expected if they have self-domesticated. Behavioural and anatomical similarities between western chimpanzees and bonobos would appear to be homoplasies, because bonobos became separated from the common ancestor in east or central Africa long before the evolution of the western subspecies (Figure 3; Prado-Martinez et al., 2013; de Manuel et al., 2016).

The possibility that three cases of self-domestication can be found in the Hominidae, a family with few living species, suggests that a wider search for cases of self-domestication will be revealing. Suggestive evidence has been found in urban vs. rural populations of red foxes, *Vulpes vulpes* (Parsons et al., 2020), and in wild marmosets, *Callithrix jacchus* (Ghazanfar et al., 2020). The evolution of dogs from wolves, *Canis lupus*, is widely assumed to have begun with self-domestication (Coppinger & Coppinger, 2000). Other possible taxa that have been speculated to be self-domesticated based on low levels of within-species aggression include some island forms compared with continental ancestors (Hare et al., 2012; Hare, 2017; Wrangham, 2019b).

## Self-domestication in *H. sapiens*

In the case of human evolution, self-domestication has been proposed to have happened in the Pliocene with Australopithecines, or in the early Pleistocene with *Homo erectus* (Table 1). Such claims may be correct but are hard to assess, so I restrict discussion to *H. sapiens*.

**Table 1.** Proposed occurrences of self-domestication in the human lineage. The case discussed in this paper is in the bottom row

Time	Evidence	Reference
4–7 ma	Bipedality, reduced canines	Raghanti (2019)
1–2 ma	Appearance of *Homo erectus*	Hare (2017)
0.1–1 ma	Brain increase	Hare (2017)
50–500 ka	Material culture	Hare (2017)
0–300 ka	Cranio-facial feminisation, cranial globularisation, skeletal gracilisation	Leach (2003), Cieri et al. (2014), Progovac & Benítez-Burraco (2019), Wrangham (2019b)

ma, Million years ago; ka, thousand years ago.

The traditional idea that humans are an unusually unaggressive species by comparison with typical wild animals has rarely been examined. In one study, the frequency of dyadic fighting among humans was two to three orders of magnitude lower than in chimpanzees or bonobos (Wrangham et al., 2006; Wrangham, 2019b). However the important comparison for understanding human self-domestication is how frequently reactive aggression occurs in *H. sapiens* compared with ancestral *Homo*, not with apes.

Injuries indicated by fossils can in theory indicate rates of fighting. Beier et al. (2018) compared fossils of *H. sapiens* with *H. neanderthalensis*, a species that has contributed around 2% of genes to living humans (Gokcumen, 2020) and which offers a helpful model of pre-*sapiens* ancestors (Williams, 2013). Based on 21 specimens that had at least one traumatic lesion in the cranium, the two species showed no difference in frequency of injury (~5% in each case), although *H. neanderthalensis* tended to be injured and die when younger. Such studies are promising, but it is difficult to distinguish injuries sustained in fighting from those incurred while hunting. Furthermore the lighter skull and skeleton of *H. sapiens* might mean that they are relatively more vulnerable to trauma. Direct evidence on the evolution of aggressiveness is therefore currently inconclusive.

Instead of direct evidence of fighting rates, the argument for self-domestication in *H. sapiens* comes from anatomical changes during evolution, comparisons with living domesticates and genetic comparisons with other *Homo* species.

First, for most of the Pleistocene, *Homo* species showed few signs of a self-domestication syndrome. Starting ~315,000 years ago, however, when the earliest fossils attributable to *H. sapiens* were formed (Hublin et al., 2017), informative trends include multiple features found in domesticated animal species. Four are used by archaeologists to recognise domestic animals in the fossil record: reduced sexual dimorphism in various bones and teeth, shorter faces and smaller molars, lighter bodies and (in the last 30,000 years) reduced cranial capacity (Leach, 2003; Cieri et al., 2014; Hublin et al., 2017). Brow ridges have also been reduced and faces have become relatively narrow. Since narrower faces nowadays are associated with reduced aggressiveness, the narrowing trend strongly suggests that *H. sapiens* has become increasingly docile (Cieri et al., 2014; Haselhuhn et al., 2015; Deska et al., 2018). In short, gracilisation tendencies similar to those found in domestication are found throughout the evolution of *H. sapiens*, and are putatively responses to selection against reactive aggression (Hare, 2017; Wrangham, 2019b). This idea contrasts with traditional views that treat each gracilised trait as responding independently to different selection pressures (e.g. Brace et al., 1987; Ruff et al., 1993, 1997; Pearson, 2000; Lieberman et al., 2002).

Second, comparisons of traits in *H. sapiens* with those in living domesticates have focused especially on dogs, *Canis familiaris*, and foxes (Hare, 2017). Compared with their wolf ancestors, dogs exhibit low reactive aggression, high play, increased cranial neoteny, high tolerance, high prosociality, low responsiveness of the HPA axis, low trabecular bone fraction, high oxytocin activity, a long juvenile period and relatively cooperative patterns of communication. All of these features are found in humans, suggesting that their occurrence is owed to a process of domestication similar to the evolution of dogs from wolves (Hare, 2017; Raghanti, 2019; Thomas & Kirby, 2018; Progovac & Benítez-Burraco, 2019; Wrangham, 2019b; Chirchir, 2021). Belyaev (1984) was inspired by these kinds of change in his experimentally domesticated foxes to suggest that humans had self-domesticated.

Third, genetic comparisons are in their infancy but are already promising thanks to a growing understanding of the mechanisms underlying domestication. A leading candidate for explaining why domesticated animals from different lineages show convergent phenotypes is a mild neurocristopathy, that is, a reduction in the rate of migration and/or total number of neural crest cells (Wilkins et al., 2014). Effects of a neurocristopathy are well established in the production of unpigmented hair at terminal sites of melanoblast migration (tips of feet, tail and forehead), a common feature of domesticated animals (San-Jose & Roulin, 2020). In other cases of domestication-linked traits, such as floppy ears, short face, reduced brain size and increased tameness itself, the putative effects of neurocristopathy are plausible but unproven (Wilkins et al., 2014; Wilkins, 2017).

The neurocristopathy hypothesis is sufficiently suggestive, however, that researchers have looked for genomic evidence to test it. Supportive evidence has been found in cats, *Felis silvestris catus* (Montague et al., 2014), rabbits, *Oryctolagus cuniculus* (Carneiro et al., 2014), horses, *Equus caballus* (Librado et al., 2017), dogs, *Canis familiaris* (Pendleton et al., 2018), rats, *Rattus norvegicus* (Singh et al., 2017), buffalo, *Bubalus bubalus* (Luo et al., 2020), and camels, *Camelus dromedarius* and *C. bactrianus* (Fitak et al., 2020). These studies suggest that domestication is often achieved at least partly by the evolution of a mild neurocristopathy.

Evidence for neurocristopathy has therefore been looked for in *H. sapiens* compared with *H. neandertalensis* and *H. denisova*, two close relatives that exhibit no anatomical signals of self-domestication. In support of the self-domestication hypothesis, neural crest-related changes are found in *H. sapiens* compared with its congeners (Theofanopolou et al., 2017; Zanella et al., 2019). Functional tests by Zanella et al. (2019) showed that reduced activity of the regulatory gene BAZ1B in *H. sapiens* causes down-regulation of multiple genes regulating neural crest migration and maintenance. As a result neural crest cells are formed and migrate more slowly in *H. sapiens* than they would have done in *H. neandertalensis* and *H. denisova*, with changes in the craniofacial structures and temperament of *H. sapiens* that are as expected from self-domestication.

Such studies should eventually include other mechanisms suspected to contribute to domestication, including changes to the thyroid hormone system and the glutamate signalling system. The thyroid hormone hypothesis proposes that tameness is essentially a juvenile characteristic that has been retained into adulthood (Crockford, 2002). Because thyroid hormones regulate growth rate, they are thought to be master regulators of domestication. The glutamate receptor hypothesis focuses on how changes in neurotransmission might contribute to a reduction in the propensity for reactive aggression, such as through attenuation of the HPA stress response and/or release of oxytocin and vasopressin (O'Rourke & Boeckx, 2020). Both hypotheses predict that domesticated and self-domesticated species will show changes in relevant genes compared with wild ancestors or sister species that have not been tamed (Wilkins, 2017).

These mechanisms, and possibly others, mean that wherever suitable comparands occur, hypotheses of self-domestication are testable using *a priori* predictions of specific genetic changes. In the case of *H. sapiens*, the split from *H. neandertalensis* and *H. denisova* is estimated to have occurred between 400,000 and 700,000 years ago (Prüfer et al., 2014; Stringer, 2016). As expected, comparisons of *H. sapiens* with those sister species indicate selection for genes related to self-domestication around 300,000 to 500,000 years (Zanella et al., 2019; Andirkó et al., 2021).

## Comparison of within-group killing in humans and chimpanzees

Evidence that the most recent evolutionary phase of human self-domestication started with *H. sapiens*, whereas earlier Pleistocene *Homo* species showed no signs of a self-domestication syndrome, suggests that the period shortly before 300,000 years ago marked the beginnings of intensified selection against reactive aggression. Subsequent acceleration of gracilisation trends from archaic to modern *H. sapiens* suggests that self-domestication has continued to the present at an increasing rate. A critical question therefore is: what happened before 300,000 years ago that would explain why reactive aggression was selected against much more strongly than previously?

Based on Boehm's execution hypothesis, the answer might seem to be that this was the first time that human ancestors developed an ability to safely kill alpha males. While I will argue that this answer is correct, it is also inadequate because other species are also known to kill adults in their own group. The problem is particularly severe for chimpanzees because, according to Boehm (2018), chimpanzees have a human-like capacity to remove a disliked despotic male by killing him. Boehm reported that in chimpanzees and humans ‘bullies … are singled out for lethal attacks’ [by coalitions] (Boehm, 2018, p. 693). Therefore, ‘It is very likely that our chimpanzee-like ape ancestors ganged up on disliked individuals to temporarily or permanently eliminate them from the group’ (Boehm, 2017, p. 763).

Those claims might be taken to suggest that there is no important difference between the propensities of chimpanzees and humans to kill an alpha male. Yet domineering behaviour is an invariable feature of alpha male chimpanzees, so it has not been reduced in that species as it has in humans. This suggested to Boehm (2017, p. 769) that there is a feature of human coalitions that makes them into ‘a special, social selection force’. The critical question can therefore be reformulated: do differences in the nature of within-group killing of adults explain why, by 300,000 years ago, reactive aggression was selected against in humans but not in chimpanzees?

The sole data available for answering this question come from Boehm's (2017, 2018) assembly of 13 relatively severe within-group gang attacks by chimpanzees on adult males (Table 2). Three of these offer very little behavioural data because they were described only from the aftermath (former alpha males Foudouko and Ntologi (in 1995), and young male Zesta). Furthermore it is not even certain that Ntologi's 1995 death was caused by chimpanzees (Nakamura & Itoh, 2015). In seven cases the victim died immediately or within days. In the other six cases the victim remained in the group with a reduced dominance rank, sometimes after a prolonged period of being solitary.

**Table 2.** Violent within-group gang attacks among chimpanzees

Victim and date	Victim social rank	Seen	How attack started	Who joined who	Delay before attack (min)	No. male attackers in first fight	No. male attackers total	Reports of V being helped?	Reports of attackers wounded?	Victim's fate	First attacker's fate	Study site, group, ref.
Pimu, 2 October 2011	Alpha	All	Dyadic fight attracted attackers	A	0	4 (incl. Alofu)	5	Yes	Yes	Dead. Fight took >2 hours	Alofu became alpha	1
Vincent, 22 December 2004	Ex alpha	All	V, badly injured, joined two meat-eating males	?	0	1 (Rudi, alpha)	2	?	No	Dead	Rudi stayed alpha	2
Grappelli, 29 October 1995	Low rank	All but start	Screams attracted alpha BT and others. Some did not attack	V?	≥6	?	7 main attackers. Alpha BT briefly involved at start	No (but some embraces)	No	Died ≥7 hours later	First attacker not known	3
Hatari, 17 February 2011	Low rank	All but start	?	?	?	1 or 2, including alpha Brutus	2	No	No	Died 10 days later	First attacker not known but Brutus stayed alpha	4
Foudouko, 15 June 2013	Ex alpha	Body only	?	?	?	?	5	No	1 male had superficial wounds	Dead	First attacker not known	5
Zesta, 4 November 1998	Low rank	Body only	V found prone 50 minutes after screams heard	?	?	?	≥3, + alpha Duane who ate ZE's flesh	No	3 males had superficial wounds	Died ~2 hours later	First attacker not known	6
Ntologi, 14 November 1995	Ex alpha	Body only	?	?	?	?	Many?	No	No	Dead	First attacker not known	7
Goblin, 25 October 1989	Ex alpha	All	V, in poor health, joins group for first time in 27 days	V	≥11	1 (alpha, Wilkie)	≥6	No	No	Exile	Wilkie stayed alpha	8
Jilba, 7 October 1991	Mid-rank	All	V joins meat-eating group (6 M, 2 F). Alpha Kalunde chased V	V	≥2	1 (alpha, Kalunde)	8	No	No	Exile 50 days	Kalunde stayed alpha	9
Sheldon, early December 2004	Ex alpha	All	V, after absence, was attacked and briefly ‘pinned to the ground’ by ≥3 M incl. alpha Kris	?	?	?	3	No	No	Exile	First attacker not known but Kris stayed alpha	10
Ntologi, 20 August 1991	Ex alpha	All	Chased by new alpha Kalunde and others	?	?	1 or more, incl. alpha Kalunde	6?	No	No	Exile	Stayed alpha	11
Ferdinand, 1 October 2016	Alpha	All	V, after long absence, arrived in good health	V	After V charged	1 (Fudge)	3. Other males watched and displayed	No	No	Lost rank to Fudge	Became alpha	12
Frodo, 27 January 2003	Alpha	All	V, in poor health, arrived in group. He was charged and attacked, mostly by beta male Sheldon	V	?	?	10? One M (brother) did not attack	Yes, F (mother)	No	Lost alpha rank to Sheldon	First attacker not known	13

Attacks are taken from Table 20.1 in Boehm (2017) and Table 1 in Boehm (2018). Some details have been added, and corrections have been made in the light of new information. Cases are ordered by whether victim died, was exiled or remained in the group. Within those categories, better observed cases are listed first.

‘Seen’: ‘All’, entire interaction observed; ‘All but start’, the attack was seen except for how it began; ‘Body only’, the attack was not seen, but the victim's body was informative.

‘How attack started’: V, victim; ?, attack not seen; F, female; M, male.

‘Who joined who’: A, aggressor joined victim; V, victim joined aggressor; T, aggressor and victim were socialising in party before the attack.

‘Delay before attack’ shows how many minutes elapsed between the antagonists being in the same subgroup and the attack starting.

‘No. male attackers in first fight’ refers to the attack that became coalitionary.

‘No. male attackers total’, Number of males recorded as participating in the attack at some point, not necessarily all at once.

‘Reports of V being helped?’: V, victim; yes, victim was defended by at least one male (M) and/or at least one female (F); no, no report of victim being defended.

‘Reports of attackers wounded?’: ‘No’, no reports of attackers being wounded.

‘Study site, group, ref.’: 1, Mahale, M-group (Kaburu et al., 2013); 2, Gombe, Mitumba (Wilson et al., 2005; Mjungu, 2010); 3, Kibale, Ngogo (Watts, 2004); 4, Kyambura, only group (Nicole Simmons, personal communication by email); 5, Fongoli, only group (Pruetz et al., 2017); 6, Budongo, Sonso (Fawcett and Muhumuza, 2000); 7, Mahale, M-group (Nishida, 1996; Nakamura & Itoh, 2015); 8, Gombe, Kasekela (Goodall, 1992; Boehm, 2017); 9, Mahale, M-group (Nishida et al., 1995); 10, Gombe, Kasekela (Wilson et al., 2004); 11, Mahale, M-group (Nishida et al., 1995); 12, Gombe, Kasekela (Mjungu & Collins, 2016); 13, Gombe, Kasekela (Fallow, 2003).

Boehm was impressed by the similarities between these within-group gang attacks and the better-known between-group gang attacks. For example he referred to ‘chimpanzees ganging up on a high-ranking community member and attacking him as strangers are routinely attacked’ (Boehm, 2018, p. 689). Within-group gang attacks indeed resemble between-group attacks in that victims are rendered helpless by the coordinated aggression of two or more males. However, several important differences suggest that, whereas between-group gang attacks can involve collective proactive aggression aimed at damaging a random stranger, within-group gang attacks involve mostly reactive aggression used opportunistically by an individual male to compete for status, aided by a supporting set of subordinates. The differences are crucial.

First, in within-group attacks no hunting behaviour has been reported. When chimpanzees raid into neighbouring territories, in contrast, they tend to show various kinds of hunting behaviour, including sniffing the ground, stopping and listening in the direction of the neighbouring group, maintaining high vigilance, and when a potential victim is detected, silently stalking before making a sudden violent attack (Wrangham, 1999; Watts, 2004).

Second, when within-group attacks were seen from their beginning, in four of the five cases the victim joined the aggressor(s) (Table 2). In between-group attacks, in contrast, the overwhelming pattern is for aggressors to arrive at the victim. The difference indicates that within-group attacks were less proactive than between-group attacks.

The exceptional incident, when a coalition arrived at the victim and immediately attacked him, was the death of Pimu (Table 2). Even this case shows little evidence of proactivity, because the attack was considered to be ‘best explained as an opportunistic challenge for social dominance’ (Kaburu et al., 2013, p. 794). It began in a relaxed social setting. Alpha-male Pimu was grooming with a rival, Primus, when for no obvious reason Pimu bit Primus on his hand. Primus responded by biting Pimu's face. The exchange of bites precipitated an intense and noisy one-on-one fight which two males tried to quell by attempting to separate the antagonists. Nine minutes after the fight began Primus had left, but unfortunately for Pimu, who by now was wounded, four males arrived and immediately attacked him. Male Alofu led these attacks, which continued intermittently for more than two hours. Despite being defended to some extent by two males, Pimu died and Alofu became the new alpha male. Apparently Alofu had heard Pimu fighting and arrived with the intention of taking advantage of his temporary weakness. This case is one of only two in which an attack started as soon as a coalition met the victim (the other being Rudi's attack on Vincent; M. Wilson, pers. comm.).

Third, within-group attacks sometimes began several minutes after the antagonists met. The timing was reported in five cases (Table 2). In three (Jilba, Grappelli and Goblin) the attack started from at least 2 to at least 11 minutes after the victim and aggressors came together. In contrast, between-group attacks invariably begin as soon as a victim is reached (Wrangham, 1999; Watts et al., 2006).

Fourth, the size of the gang reported in within-group attacks has been smaller than that in between-group attacks. In two of the seven lethal attacks listed in Table 2 there were only two aggressors, and of the total of 11 gang attacks, more than half had five attackers or fewer. In contrast the smallest number of males recorded in 10 lethal between-group attacks on adult males listed by Wilson et al. (2014) was three, which was the only case in which fewer than six attackers were involved. This indicates that in within-group interactions, the power imbalance was relatively less, suggesting that attacks were riskier and more costly.

Fifth, in two within-group attacks the victim was intermittently defended: Pimu was supported by allied males, and Frodo was supported by his mother, who received some superficial wounds as a result. Injuries to the aggressors were also reported in three cases (Table 2). In between-group attacks, in contrast, victims are undefended and immobilised too completely to effectively fight back.

Sixth, within-group attacks could continue intermittently for much longer than the typical between-group attack (up to more than 2 hours, compared with 10–20 minutes for between-group attacks; Kaburu et al., 2013). The aggressors could afford to be relaxed because they were in familiar territory where there was little chance of being surprised by members of other communities, unlike the typical context of between-group encounters.

Seventh, within-group attacks showed a strong tendency to be related to within-group tensions incurred by competition for the alpha rank. In at least three cases the fight began with the alpha male attacking on his own (victims Vincent, Goblin and Jilba). Three times out of a total of 10 cases the alpha lost: he was killed once (Pimu) and lost his alpha rank twice (Ferdinand, Frodo). In those events the attacks were led by the male who then became alpha. Seven times the alpha won: the alpha was a main aggressor in three fights in which the victim died (Vincent, Hatari and Zesta), and in four further cases in which the victim went into exile (Goblin, Jilba, Sheldon and Ntologi, August 1991). Between-group attacks, in contrast, have not been reported to have any implications for the alpha male's status.

In chimpanzees, the male whose bullying is most intense is invariably the alpha male: he forces all others to give frequent signals of submission (Goodall, 1986). If bullies were being ‘singled out for lethal attacks’, therefore, alphas should have been the most frequent victims. That alphas were attackers more often (six times) than they were victims (three times) clearly undermines that account. Alpha males are under frequent pressure to defend their rank by defeating challengers in physical fights. Equally, challengers are constantly looking for opportunities to confront the alpha in an advantageous context. Notably, Table 2 shows that the male who became or continued as alpha after the interaction was invariably the individual who led the gang attacks. Thus chimpanzee gang attacks favoured the ‘bullies’, whether they were aspiring or incumbent alphas. This pattern is opposite to the reversed dominance hierarchy described after human within-group killings.

In sum, compared with the premeditated nature of between-group gang attacks, to date within-group attacks show less or no evidence of planning, less immediacy, less one-sidedness, fewer attackers, higher risk of being wounded and longer duration. Overall within-group attacks appear to be less efficient and less organised than between-group attacks. Current evidence does not support bullies being ‘singled out for lethal attacks’.

Chimpanzee within-group attacks are also less efficient and less planned than human within-group killings. Hunter–gatherer styles of within-group killing are well known. Most often an executioner, typically a kinsman of the victim, is delegated in advance and kills by ambush. Alternatively a group unites to make a coordinated physical attack, or a killer's action are approved by the community after he takes sole responsibility for killing a disliked individual. The kills are cheap and effective not only because lethal weapons are used, but also because the community agrees that a specific victim should be killed or deserves to die (Boehm, 1999, 2017). Without such agreement an executioner risks being regarded as a danger to other group members, and therefore becoming vulnerable to being killed himself. Planning is thus more evident in human executions than in chimpanzee within-group gang attacks.

Taken together, the limited available data show critical similarities and differences in within-group gang attacks among chimpanzees and humans. The two species are similar in using coalitions that can assemble immense power to kill or overcome a victim. Chimpanzee within-group gang attacks differ from human executions, however, by showing little or no sign of being planned, and no consistent ability to kill the alpha male. Instead, they appear to develop spontaneously as reactions to various contexts including aggression by an alpha male towards a rival, aggression by a rival towards an alpha male and a resented aggressor being physically weak and/or in the process of being defeated. Most important, chimpanzee within-group gang attacks differ from human executions because they are not levelling mechanisms.

These conclusions suggest that differences in the features of within-group gang attacks between humans and chimpanzees can indeed explain why reactive aggression is selected against in humans but not in chimpanzees. In both species subordinate males would appear to benefit from escaping the domination of an alpha male. Human subordinates can achieve that goal, because they can create coordinated plans to safely kill even the most individually intimidating member of their group. In contrast chimpanzees cannot create such plans. Being unable to predictably eliminate a despotic rival, their behaviour does not create a selection pressure against the bullying behaviour characteristic of alpha males.

## Discussion

### Why within-group gang attacks have not favoured groupishness in chimpanzees

A main aim of this review has been to investigate why, if the execution hypothesis is correct, self-domestication and groupishness have not been selected by within-group killing among chimpanzees. Two differences between human executions and chimpanzee within-group gang attacks appear to be critical.

First, the coalitionary aggression used by chimpanzees in within-group gang attacks is constrained to being largely reactive, whereas human executions are mostly proactive. Proactive gang aggression by chimpanzees is possible only against members of other groups, when all out-group members are candidate victims for all raiders. In those circumstances no discussion is needed to determine who sides with whom, or who the target is. Within groups, in contrast, a shared plan would be necessary to identify a potential victim and assemble a coalition, but the limited ability of chimpanzees to share intentions means that no such plan is possible. This means that, unlike humans, chimpanzees cannot systematically victimise antisocial individuals.

Second, chimpanzee within-group killings maintain the alpha-male role, whereas human within-group killings eliminate it. Chimpanzee attacks are typically led by individuals who are defending their alpha status or attempting to acquire it. Other members of a coalition conform to a ‘winner-support’ strategy (Nishida et al., 1995). In humans living in small-scale, acephalous societies, in contrast, executions are levelling mechanisms: they are used to stop anyone from behaving despotically. Admittedly large-scale human societies have leaders, but those leaders are not alphas in the animal style. Unlike animals, human leaders depend for their power on the strength of their coalitions rather than their ability to defeat rivals in one-on-one fights. The apparent similarities in within-group killings among chimpanzees and humans are thus deceptive. Chimpanzees are limited to reactively targeting rivals for alpha male status, whereas humans can proactively eliminate alpha males.

### The importance of targeted conspiratorial killing

Targeted conspiratorial killing is importantly different from less coordinated styles because, by allowing a plan to be made, it can make the costs of killing exceptionally low even when the victim is an individually intimidating fighter. Advanced weaponry is expected to contribute to reducing the costs of lethal aggression but it is not vital, as shown by the kills made in between-group attacks by chimpanzees, wolves and other species (Wrangham, 1999). The vital factor enabling proactive killing within groups is the ability to conspire in a way that permits coalition partners to identify a victim, develop an efficient plan and carry it out so as to give the killers a massive tactical advantage. After that ability had evolved, humans became subject to a new form of social selection that no previous vertebrate had experienced, namely to be less aggressive, less antisocial, more conformist and more punitive of non-conformism than before, lest they be deliberately killed.

A focus on targeted conspiratorial killing thus builds on Boehm's execution hypothesis by proposing that a uniquely human ability was a necessary condition for developing human groupishness. Self-domestication, according to Boehm's hypothesis, was the first consequence of targeted conspiratorial killing, when subordinate males collaborated to kill the alpha. The coalition of males who thus held bullies in check then became a power over the group, able to impose a collective will on all types of non-conformism and thereby inadvertently selecting for multiple psychological traits that underlie different facets of human groupishness.

### The role of language

Human self-domestication and language have been proposed to be related to each other by a positive feedback loop. Self-domestication leads to less fighting and more tolerance. The increase in inter-individual tolerance allows more effective use of language, which leads to coordination becoming more skilled. Groups can then better suppress bullies, whether through more effective verbal aggression (Progovac & Benítez-Burraco, 2019; Del Savio & Mameli, 2020), social ostracism (Boehm, 2012; Del Savio & Mameli, 2020) or killing (Boehm, 2012; Wrangham, 2019b). Equivalent suggestions have been made for the relationship between groupishness and language (e.g. Boehm, 2012). Such ideas are not controversial, although they have not been much elaborated.

The relationship between self-domestication and language is more contested with regard to origins. One kind of proposal holds that the evolution of language depended crucially on self-domestication (Thomas & Kirby, 2018; Progovac & Benítez-Burraco, 2019). If so, targeted conspiratorial killing could not have been responsible for initiating self-domestication. Alternatively, as implied in this paper, language became sufficiently sophisticated before the origin of self-domestication that it launched the process by making targeted conspiratorial killing possible (Wrangham, 2019a, b). The difficulty about that idea is that it requires an explanation for how language became sophisticated before self-domestication kicked in. Such an explanation would need to include why the ancestors of *H. sapiens* developed a more sophisticated language than other *Homo* species (especially *H. neanderthalensis* and *H. denisova*) that do not show anatomical signs of self-domestication. No such explanations have apparently been proposed.

### Objections to the putative role of targeted conspiratorial killing in favouring self-domestication and groupishness

One kind of objection to the execution hypothesis is that it is not needed because alternative ideas can explain the evolution of self-domestication and/or exceptional groupishness. Such alternatives include two major classes.

The first comprises hypotheses that explain how the benefits of groupish behaviour could have been favoured. For instance Tomasello et al.'s (2012) interdependence hypothesis suggested that selection favoured individuals who cooperated to solve newly intense ecological problems. Boyd and Richerson (2005) argued that selection among cultural systems led to the most cooperative groups succeeding. Sober and Wilson (1998) argued that prosocial moral systems evolved by group selection.

The second comprises hypotheses that focus on the control of aggression, at least as an initial step. Hare (2017) proposed that as brain size increased, there was a coincident increase in self-control that permitted inhibition of aggressive tendencies (cf. Shilton et al., 2020). Del Savio and Mameli (2020) suggested that increasing linguistic skills allowed group members to ostracise antisocial individuals so effectively that aggressive tendencies would be selected against. Gleeson and Kushnick (2018) argued that females could favour reduced aggression in males by choosing less aggressive males as mating partners.

A challenge for both types of explanation is to understand how selection would have caused alpha males to have reduced fitness (Wrangham, 2019a). By definition, an alpha male in a small group can outcompete others for access to resources. Hypotheses therefore need to explain not only why benefits accrue to the less aggressive, but also why despotic males could not obtain those benefits by physical force. Some such explanations have been advocated. Del Savio and Mameli (2020) argued that domineering individuals could be excluded from resources by being socially ostracised rather than killed. Whether social ostracism or similar lesser punishments can be an effective force in reducing fitness without being backed up by an ultimate threat of execution, however, has to my knowledge not been demonstrated. Furthermore killing a bully may be less costly than non-lethal punishment, since killing reduces the risk of the victim fighting back, whether immediately or in the future. Gavrilets (2012) modelled a related idea, which was that alpha males predictably lost resources to coalitions that used reactive rather than proactive aggression. His model assumed that helpers supported victims despite such fights being relatively costly. Application of such models to proactive lethal attacks is desirable.

The proposal that targeted conspiratorial killing had a major influence in promoting human groupishness does not rule out the possibility that important elements of groupishness had already been present in pre-*sapiens* species of *Homo*. Such ancestral elements could have facilitated food-sharing among non-kin, a sexual division of labour, and the development of an initial form of language, for example. According to the execution hypothesis as presented here, however, the development of targeted conspiratorial killing represented a critical advance because it alone explains the replacement of an ordinary alpha-male hierarchy with a reverse dominance hierarchy, with its many consequences for prosociality (Boehm, 1999; Wrangham, 2019a).

## Data Availability

No original data are presented.
